# Review of methodology and re‐analysis of lipidomic data focusing on specialised pro‐resolution lipid mediators (SPMs) in a human model of resolving inflammation

**DOI:** 10.1111/iep.12523

**Published:** 2024-12-23

**Authors:** Natalie Z. M. Homer, Ruth Andrew, Derek W. Gilroy

**Affiliations:** ^1^ Mass Spectrometry Core, Edinburgh Clinical Research Facility University/BHF Centre for Cardiovascular Sciences, Queen's Medical Research Institute Edinburgh UK; ^2^ University/BHF Centre for Cardiovascular Science, Queen's Medical Research Institute, University of Edinburgh Edinburgh UK; ^3^ Department of Ageing Rheumatology and Regenerative Medicine, Division of Medicine, The Rayne Building, University College London UK

**Keywords:** inflammation, lipidomics

## Abstract

Using a model of UV‐killed *E. coli* driven dermal inflammation in healthy human volunteers, we originally reported that following inflammatory resolution there was infiltration of macrophages, which, through prostanoids including prostaglandin (PG) E_2_, imprints long‐term tissue immunity. In addition to the prostanoids, data on levels of Specialised Pro‐Resolution Lipid Mediators (SPMs) throughout inflammatory onset, resolution and post‐resolution phases of this model were presented, but as illustrations rather than as primary data. Therefore, in response to a request for increased transparency, a subset of the original data from our human UV‐killed *E. coli* model was re‐analysed by two experts from an independent laboratory alongside a review of the methodology used. The prostanoids were detected robustly following re‐analysis but the areas of the chromatographic peaks of the SPM lipid mediators were too small to yield amounts that could be reliably detected and/or quantified using community standards. Importantly, with prostanoids including PGE_2_ being robustly detected, this re‐analysis does not alter the original report that post‐resolution PGs imprint tissue immunity. Here we show the outcome of this re‐analysis and review the biology surrounding SPMs as a result.

## 1 BACKGROUND

We originally examined the role of lipid mediators in the resolution and post‐resolution phases of an experimental model of self‐limiting inflammation in healthy human volunteers triggered by an intradermal of UV‐killed *E. coli* (UV‐KEc).^1^ This necessitated the use of mass spectrometry (MS) as the gold standard technique, combined with liquid chromatography (LC) to determine the presence and quantities of lipid mediators in inflammatory exudates that were extracted from the inflamed skin. These lipids included *S*pecialised Pro‐Resolution Lipid Mediators (SPMs), prostaglandins (PGs) and leukotrienes (LTs).

However, an external party raised a concern regarding an illustration used to depict lipid molecules identified in samples displayed in fig. S1 of the above paper. This was in support of concentrations and temporal profiles of a representation of these lipids presented in fig. 4A–H. The concern was raised that such illustrations in fig. S1 might be misunderstood as reporting raw data. In response, an unbiased re‐analysis of raw data from a subset of samples was conducted, in accordance with harmonised ICH guidelines^2^, ^3^ and by two experts from an independent laboratory. The aim was to replace the “illustration” with primary data in a manner aligned to open scientific principles and presented in a manner that could be critiqued by other MS experts while being understood by non‐MS experts who have an interest in inflammation, resolution and lipid biology.

A subset of the raw data (detailed in ‘Outcome of re‐analysis’ section) was provided and upon re‐analysis the integrated areas of the chromatographic peaks of many of the SPM lipid mediators previously represented in the Supplementary file and fig. 4 were found to be less than areas that could be reliably either detected and/or quantified using signal to noise ratios of 3:1 and 10:1, respectively, as indicative respective cut‐offs for limits of detection and quantitation, respectively; these settings reflect community standards for quantitation [2]. This re‐analysis presents the revised findings.

## 2 RE‐ANALYSIS PIPELINE

Originally, samples of blister fluid were collected from nine time points following bacterial injection including naïve skin.^1^ fig. 4 of the original paper presented data on prostanoids (PGE_2_, PGD_2_, PGF_2_α and TxB_2_), and limited numbers of SPMs (LxB4, RvD3 and RvD5), and data in the accompanying supplementary fig. S1A comprised illustrations of additional members of the SPM family.

Though the full dataset was requested, only a subset of datafiles were provided which were re‐assessed to derive abundances of a representative number of the SPM family, including those in fig. 4 and in Supplementary fig. S1A namely RvD1; 17R‐RvD1; RvD3; 17(R)RvD3; RvD4; RvD5; RvD6; 10S,17S‐diHDA; PD1; MaR1; 7S,14S‐diHDHA; RvE1; RvE2; RvE3; LXA4; LXB4; 15‐epiLXA4 and 5S,15S‐diHETE. Data pertaining to PGE_2_, PGD_2_, PGF_2α_ and TxB_2_ as well as LTB_4_ were also reanalysed. The subset of biological samples included blister fluid collected at 8 h (early inflammation onset) and at 48 h (resolution) post‐UV‐KEc injection of the original analysis; these time points represented when pro‐resolution entities would most likely be synthesised.

**FIGURE 1:**
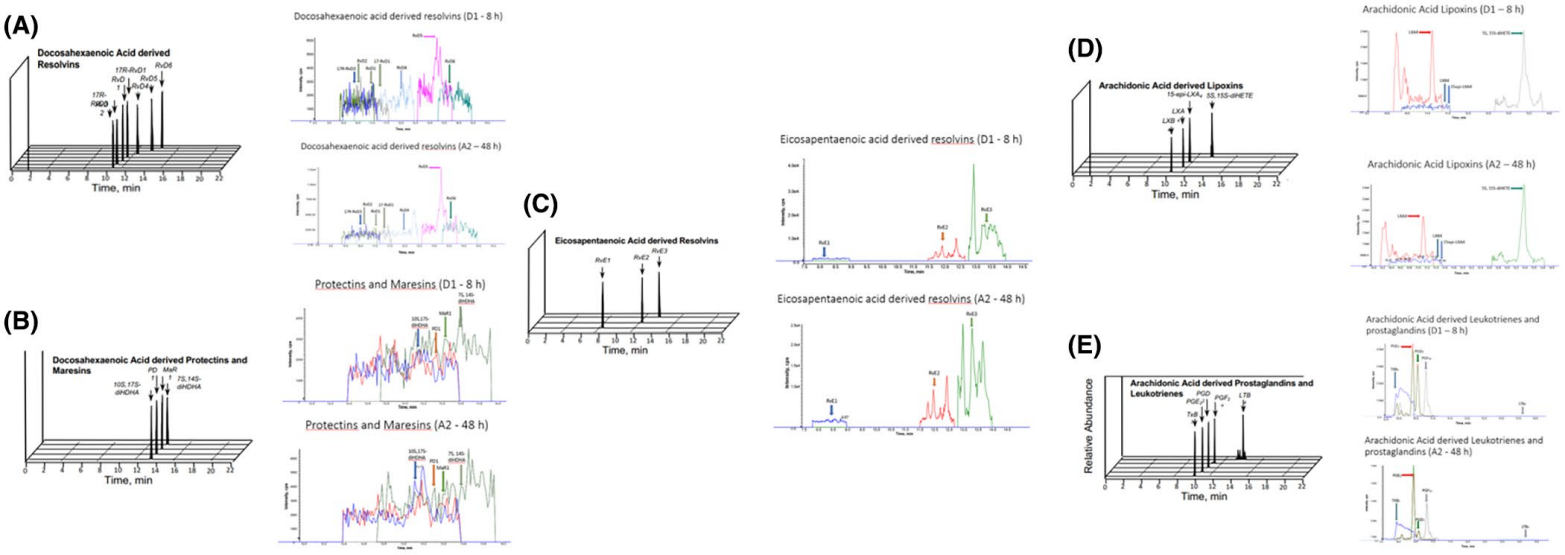
Lipid mediator profile displayed in five lipid mediator groups (A–E) as in Madhur et al., *PLoS*, 2017,^1^ compared alongside overlaid extracted ion chromatograms of an 8‐h (D1) and 48‐h (A2) timepoint sample, extracted using Analyst® version 1.7 software, according to retention times and mass transitions (*m/z* parent—product ion). (A) Docosahexaenoic acid‐derived resolvins: 17(R)RvD3 (RT = 10.47 min) *m/z* 375.2 ➔ 137.0, RvD3 (RT = 10.64 min) *m/z* 375.2 ➔ 137.0, RvD1 (RT = 11.08 min) *m/z* 375.2 ➔ 141.1, 17R‐RvD1 (RT = 11.25 min) *m/z* 375.2 ➔ 215.2, RvD4 (RT = 11.99 min) *m/z* 375.2 ➔ 101.0, RvD5 (RT = 13.20 min) *m/z* 359.2 ➔ 199.2, RvD6 (RT = 13.70 min) m/z 359.2 ➔ 215.2. (B) Docosahexaenoic acid‐derived protectins and Maresins: 10S,17S‐diHDA (RT = 13.10 min) *m/z* 359.2➔181.1; PD1 (RT = 13.30 min) *m/z* 359.2 ➔ 153.1; MaR1 (RT = 13.40 min) *m/z* 359.2➔ 141; 7S,14S‐diHDHA (RT = 13.60 min) *m/z* 359.2 ➔ 141.1. (C) Eicosapentaenoic acid‐derived resolvins: RvE1 (RT = 8.15 min) *m/z* 349.2 ➔ 161.2, RvE2 (RT = 11.90 min) *m/z* 333.3 ➔ 159.2; RvE3 (RT = 13.40 min) *m/z* 333.3 ➔ 251.3. (D) Arachidonic acid‐derived lipoxins: LXA4 (RT = 11.10) *m/z* 351.1 ➔ 115.1; LXB4 (RT = 10.59 min) *m/z* 351.1 ➔ 221.1; 15‐epiLXA4 (RT = 11.36 min) *m/z* 351.1 ➔ 115.1; 5S,15S‐diHETE (RT = 13.30 min) *m/z* 335.3 ➔ 115.1. (E) Arachidonic acid‐derived prostaglandins and leukotrienes: TxB_2_ (RT = 9.91 min) *m/z* 369.3 ➔ 169.1; PGE_2_ (RT = 10.39 min) *m/z* 351.1 ➔ 189.1; PGD_2_ (RT = 10.50 min) *m/z* 351.1 ➔ 189.1; PGF_2a_ (RT = 10.78 min) *m/z* 353.3 ➔ 193.1; LTB_4_ (RT = 13.6 min) *m/z* 335.3 ➔ 195.1. RT, retention time; 5S,15S‐diHETE‐5S,15S‐dihydroxyeicosatetraenoic acid; 7S,14S‐diHDHA, 7S,14S‐dihydroxydocosahexaenoic acid; 10S,17S‐diHDHA, 10S,17S‐dihydroxydocosahexaenoic acid; 15‐epiLXA4, 15‐epi‐lipoxin A4; 17(R)RvD1, 17(R) resolvinD1; 17(R)RvD3, 17(R) resolvin D3; LTB4, leukotriene B4; LXA4, lipoxin A4; LXB4, lipoxin B4; RvD1, resolvin D1; RvD3, resolvin D3; RvD4, resolvin D4; RvD5, resolvin D5; RvD6, resolvin D6; RvE1, resolvin E1; RvE2, resolvin E2; RvE3, resolvin E3; MaR1, maresin 1; TxB2, thromboxane B2; PD1, protectin D1; PGD_2_ , prostaglandin D_2_ ; PGE_2_ , prostaglandin E_2_ ; PGF_2a_ , prostaglandin F_2a_.

Retention times and several indicative mass transitions of lipid mediators used in the original LC–MS/MS methods were applied. MultiQuant 3.0.3 software (Sciex, Warrington, UK) was used to create an automated method of integration according to multiple reaction monitoring (MRM) of transitions between precursor (Q1) and product (Q3) ions (denoted Name of Transition) and retention times. This detail is described in Figure 1 legend. For all analytes the following parameters were stipulated; a retention time half window of 5.0 seconds, a minimum peak width of 3 points and a minimum peak height of 3000 or 10,000. In addition, a noise percentage of 99% and a peak splitting of only 1 point was stipulated. No manual peak picking was carried out. A 5‐s retention time threshold was defined to aid in discrimination of isobaric lipids that closely elute in this method [e.g. RvD2 with retention time of 10.51 min and RvD3 with retention time of 10.64 min (Figure 1A)]. In the absence of stable isotopically labelled (SIL) internal standards for each lipid mediator in the extracted samples and their absence from the analytical method, then restricting the peak width to 5 seconds and assuming retention time consistency across the batch are the limitations in which we worked for this reanalysis approach.

In the absence of validation data, a minimum signal‐to‐noise ratio (SNR) of three to assign an indicative limit of detection (LOD) and the second rule of SNR of 10 to assign an indicative limit of quantitation (LOQ) were applied using unsmoothed data.^3^ Baseline was assigned adjacent to, and ahead of the peak of interest in the biological samples. For example, if the average baseline was assigned a 1000 peak height then peak heights of 3000 or 10,000 were applied to indicate the LOD and LOQ, respectively.^2^, ^3^ Data from a non‐contemporaneous, representative standard curve were provided and the accuracy of the relevant range of calibration points for values was cross‐referenced again to give an indication of the limits of robust quantitation, in conjunction with peak heights of the lowest standards.

## 3 OUTCOME OF RE‐ANALYSIS

Data in Figure 1 display the outputs of re‐analysis of SPMs and SNR >10 (LOQ). Re‐analysis of the data provided for the samples did not yield detectable peaks (SNR >3) for the docosahexaenoic acid‐derived resolvins RvD1; 17R‐RvD1; RvD3; 17(R) RvD3; RvD4; RvD6 (Figure 1A). Furthermore, peaks were not detected for the protectins or maresins: PD1; MaR1, 7S, 14S‐diHDHA (Figure 1B), or the arachidonic acid‐derived LXA4, LXB4 or 15‐epi‐LXA4 (Figure 1D) or for the eicosapentaenoic acid‐derived RvE1, RvE2 or RvE3 (Figure 1C). Peaks for RvD5, and 10S,17S‐diHDHA were putatively detected, but did not reach the cut‐off SNR >10 to permit quantitation. Except for LXB4, all values of peak areas were smaller than the lowest point in the calibration curve. For LXB, four samples had peak areas between the lower and second lowest points and thus deserve further scrutiny following full validation of the LOQ. In contrast, the prostaglandins PGE_2_, PGD_2_, PGF_2α_ and TxB_2_ as well as LTB_4_ were robustly detected in all or an appropriate subset of samples (Figure 1E). The arachidonic acid‐derived lipoxin that is suspected to be 5S,15S‐diHETE (denoted 5,15‐diHETE hereafter) was detected in some samples with SNR >10, with values between the lowest and third lowest point in the calibration curve, thus again requiring full validation of the LOQ to quantify with confidence.

In summary following re‐analysis of data, quantifiable peaks of SPMs in blister fluid were not found in contrast to the original fig. 4 and Supplementary figure.^1^

## 4 DISCUSSION AND REVIEW OF LITERATURE

We showed in rodents that there is a novel phase of prolonged immune activity following resolution of the original inflammatory response that shapes long‐term tissue immunity and preserves tissue integrity.^4^, ^5^ We discovered that these events are mediated, in part, by mononuclear phagocyte‐derived PGs.^5^ The aim of the original manuscript^1^ was to translate these findings into our human model of dermal inflammation. There, we found that once inflammation in response to intradermal UV‐KEc had resolved (as defined by clearance of heat, redness, swelling and pain as well as tissue immune cells and inflammatory mediators), there was infiltration of macrophages with robust prostanoid biosynthesis. As in our rodent studies, blocking post‐resolution prostanoids using naproxen revealed a role for these lipids in shaping tissue immunity.

As part of the original lipidomic analysis,^1^ profiles of SPMs were also reported (fig. 4A–D) and a request was received for these images to be replaced with raw data. To comply, re‐analysis of these data revealed that the prostanoids were detected as per the original report and with concentrations within the range anticipated to be quantified robustly. However, peaks were not detected for many SPMs in blister fluid, examined in accordance with harmonised ICH guidelines,^2^, ^3^ and results in our conclusion that the presence of SPMs, originally presented in the Supplementary file (1) and the quantities of LXB4, RvD5 and RvE3 in fig. 4E,F,H of the original report of human inflammatory exudates traversing three phases of inflammatory onset, resolution and post‐resolution, must be ignored. Most of the values presented for quantities of 5,15‐diHETE in fig. 4G are within the range of the calibration curves but mainly at the low end of the range. Thus, without validation of lower limits of quantitation, these must be interpreted with caution.

It is normal practice to conduct full validation using ICH harmonised guidelines^2^, ^3^ with a thorough assessment of LODs and LOQs, but in the absence of full validation outcomes, some pragmatic decisions were made to derive the re‐analysis pipeline. We used SNR >3 as a global setting as cut‐off for the lower limits of detection and an SNR >10 as a cut‐off for the lower limits of quantitation. There have been recent community changes in assignment of LOQ from 5:1 to 10:1 within the EMA guidelines which aligns with our experience that peaks with SNR of 5:1 rarely provide sufficiently precise or accurate quantitation in biological matrices. Noise varies between MRMs, particularly in matrix samples and was estimated on an individual basis for each analyte visually without application of computer integration algorithms. To avoid manual bias, integration used MultiQuant 3.01.31721 software and was fully automated for all samples with subsequent visual checks. In terms of calibration, a 1/×2 weighting to enhance the quality of quantitation at the lower points in the curve but despite this, the peak areas of many analytes in the samples were smaller than the peak area of 0.78 pg. (lowest) standard, meaning it is unlikely these values could be quantified reliably. The analytes that likely could be quantified were PGE_2_, PGD_2_, PGF2α and TXB_2_ where peak areas in the sample were substantially higher than those of the lowest standard in all biological samples, LXB4 (in 4 of the 10 biological samples) and 5,15‐diHETE (in 8 of the 10 biological samples). For LXB4 and 5,15‐diHETE these data relate to low abundance peaks and rely on quantitation using the low end of the calibration range. Therefore, when working in this range of concentrations, further efforts to ensure robust validation of the lower limit of the assay would be required to be confident to compare the quantities derived. Lastly several MRMs, with careful retention time alignment, were evaluated for each lipid but this did not alter the interpretation above. Overall, there is a need for full validation of the method, committing to a consistent major MRM transition for a given matrix for quantification and qualification, with the inclusion of paired isotopically labelled internal standards and contemporaneous calibration standards wherever possible.

The community relies on robust method validation to underpin biological findings. We cannot be experts in all things and for those areas that we do have knowledge we all play our part in upholding the standards expected by the research community, funders and the public. An important outcome from this review and re‐appraisal of a subset of lipidomic data is to acknowledge that reliable quantitation of LC–MS data is entirely dependent on consistent methodology being applied with rigorous and transparent standards. Retention time and quantitative MRM transitions of each compound are the foundation of an LC–MS/MS quantitation method, and quantitative methods should be ‘locked down’ once validated and great care taken on reporting of data at the lower limits of quantitation of the method. In addition, method transfer to a new instrument requires re‐validation. As software and hardware of technologies develop and improve, then robust automated processes can be more easily included into analytical workflows avoiding personal bias and this can help community constituents to evaluate data with confidence. To enable community assessment the dataset has been provided in an open access repository (https://doi.org/10.5522/04/27620181.v1) as per Level 2 in the TOP Guidelines (https://osf.io/9f6gx/wiki/Guidelines/).

Alongside the outcome of this re‐analysis, similar concerns^6^ were raised about claims that levels of plasma SPMs from patients with early rheumatoid arthritis could predict response to biologic therapy.^7^ The concerned party reviewed the methods, supplementary analytical data, and the online peer review file of this report and raised significant doubts regarding analytical methods and experimental conclusions.^6^ They caution that the application of this flawed methodology to SPM analysis brings into question the very occurrence of many of these lipids in biological samples, their proposed impact on inflammatory processes, and claims of their utility as biomarkers. These conclusions are echoed by a similar assessment of the putative biosynthesis and signalling pathways of SPMs in various biological systems.^8^

In addition to this re‐analysis casting substantial doubt on the existence of SPMs at biologically meaningful levels in human resolving samples, there is the additional matter of whether SPMs signal their apparent effects through their putative receptors. N‐formyl peptide receptor 2 (FPR2/ALX), GPR32 and GPR18 have been described as receptors for different D‐series resolvins.^9^, ^10^, ^11^, ^12^, ^13^, ^14^ However, this could not be confirmed by independent studies.^15^, ^16^, ^17^, ^18^ Moreover, LxA_4_ and aspirin‐triggered epi‐Lxs (15‐epi‐LxA_4_) were proposed to act through ALX/FPRL1.^19^, ^20^ However, Christophe and colleagues found LxA_4_ to be without effect on WKYMVM‐induced signalling through FPRL1/FPR2—suggesting that it acts through a receptor different from FPRL1/FPR2.^21^ On this note, it is worth directing the reader to a study which investigated the safety and tolerability as well as the pharmacokinetic/pharmacodynamic profile of an FPRL1/FPR2 agonist, ACT‐389949, in a human LPS lung challenge model. Here, the FPRL1/FPR2 receptor agonist had no effect on percentages of lung macrophages or neutrophils and in fact caused a marked increase in plasma pro‐inflammatory cytokines.^22^ Hence, far from any anti‐inflammatory and/or pro‐resolution effects of FPRs as inferred from their association with SPMs, their role in the acutely inflamed human lung maybe highly nuanced.

In support of the work of Christophe and colleagues,^21^ new research casts further doubt on whether LxA4 signals through FPR2.^23^ Here, RAW 264.7 macrophages, whilst capable of synthesising LXA_4_, cannot generate detectable levels of LXA_4_ or its 15‐oxo‐LXA_4_ metabolite when supplemented with its substrate arachidonic acid, or activated by LPS. Moreover, neither LXA_4_ nor 15‐oxo‐LXA_4_ displayed ligand activity for FPR2 when compared to the *bono fide* FPR2 ligand WKYMVm. However, arising from rigorous experimentation it transpires that the scientific explanation for the hitherto putative immune regulatory effects of LxA4 is attributed to 15‐oxo‐LXA_4_ activating nuclear factor (erythroid‐related factor 2)‐like 2 (Nrf2)‐regulated gene expression of anti‐inflammatory genes and inhibited nuclear factor (NF)‐κB‐regulated pro‐inflammatory mediator expression. Importantly, in this study, LXA_4_ did not impact these macrophage anti‐inflammatory responses.^23^

More recently, while GPR32, GPR18, FPR2, GPR37 and LGR6 were shown not to be activated by SPMs,^15^, ^16^, ^17^, ^18^ such as protectins, maresins and D‐series resolvins, they were shown to function as biased positive allosteric modulators of the PGE_2_ receptor EP4.^24^ They increase PGE_2_‐induced G_s_‐mediated formation of cAMP and thereby promote anti‐inflammatory signalling of EP4. In addition, SPMs endow the endogenous EP4 receptor on macrophages with the ability to couple to G_i_‐type G‐proteins, which converts the EP4 receptor on macrophages from an anti‐phagocytotic receptor to one increasing phagocytosis, a central mechanism of the pro‐resolving activity of SPMs. Importantly, in the absence of EP4, SPMs lose their anti‐inflammatory and pro‐resolving activity in vitro and in vivo. It was concluded that the EP4‐modify effects of SPMs only occur at supra‐physiological concentrations, these events are unlikely to occur in vivo.

These findings may explain the lack of effects of RvD_3_, LXB_4_, RvD_1_ and LXA_4_ reported by us on bacterial phagocytosis in vitro and in the same report potent anti‐inflammatory effects of a cannabinoid receptor agonist in a human model of resolving inflammation at concentrations that do not modulate SPMs.^25^ Equally, in a model of chronic granulomatous inflammation in rodents, we report random effects of some SPMs on some indices of inflammation/resolution.^26^

In summary, we present these data for broader academic consideration and discussion. These revised data presented here do not detract from the role of other lipids in post‐resolution biology as published.^1^, ^5^

## CONFLICT OF INTEREST STATEMENT

The authors declare no conflict of interest.
